# Dr. J. W. Clowes

**Published:** 1900-10-15

**Authors:** 


### OBITUARY.

### DR. J. W. CLOWES.

Dr. J. W. Clowes died September 9th. Another of our veterans has fallen at seventy-nine years, over fifty of it in the actual practice of dentistry. No one in our profession has been much better known by a connection with emphasized methods of practice. He early antagonized prominent practitioners by his advocacy of amalgam, for the feeling at that time ran high against it, yet the material has survived and to-day is second only to gold as a filling material. No practitioner ever did more to make it respectable, and this was accomplished by his conscientiousness in the handling of it. Only those who have seen specimens of his untiring industry in this line can have any idea of his work. He had the courage of his convictions, and adhered firmly to same. His patronage has been large, and principally among well to-do people who thoroughly appreciated his services. His most notable characteristic was his geniality.

Dr. Clowes began the study of dentistry in 1838 in the office of Dr. J. Smith Dodge, his brother in-law. The following year he entered the Baltimore College of Dental Surgery, and was the last surviving member of the first class graduated by that institution.

In 1842 he opened an office in New London, Conn., and remained there until he was, as he used to say, "starved out," when he went to Columbus, Ga., which gave an excellent practice for several years. In 1850, like many others, even to this day, he came to New York, and here is where he battled with the amalgam haters. When Dr. Atkinson came to New York Dr. Clowes was from the start one of his most enthusiastic admirers, and he even took an office in his house so that he might become more familiar with Atkinson's advanced ideas.—*New York Letter in Dental Digest.*

